# Astaxanthin Alleviates Ochratoxin A-Induced Cecum Injury and Inflammation in Mice by Regulating the Diversity of Cecal Microbiota and TLR4/MyD88/NF-*κ*B Signaling Pathway

**DOI:** 10.1155/2021/8894491

**Published:** 2021-01-05

**Authors:** Yueli Chen, Shiwei Zhao, Danyang Jiao, Beibei Yao, Shuhua Yang, Peng Li, Miao Long

**Affiliations:** Key Laboratory of Zoonosis of Liaoning Province, College of Animal Science & Veterinary Medicine, Shenyang Agricultural University, Shenyang 110866, China

## Abstract

Ochratoxin A (OTA) is a common environmental pollutant found in a variety of foods and grains, and excessive OTA consumption causes serious global health effects on animals and humans. Astaxanthin (AST) is a natural carotenoid that has anti-inflammatory, antiapoptotic, immunomodulatory, antitumor, antidiabetes, and other biological activities. The present study is aimed at investigating the effects of AST on OTA-induced cecum injury and its mechanism of action. Eighty C57 mice were randomly divided into four groups, including the control group, OTA group (5 mg/kg body weight), AST group (100 mg/kg body weight), and AST intervention group (100 mg/kg body weight AST+5 mg/kg body weight OTA). It was found that AST decreased the endotoxin content, effectively prevented the shortening of mouse cecum villi, and increased the expression levels of tight junction (TJ) proteins, consisting of occludin, claudin-1, and zonula occludens-1 (ZO-1). AST increased the number of goblet cells, the contents of mucin-2 (MUC2), and defensins (Defa5 and *β*-pD2) significantly, while the expression of mucin-1 (MUC1) decreased significantly. The 16S rRNA sequencing showed that AST affected the richness and diversity of cecum flora, decreased the proportion of lactobacillus, and also decreased the contents of short-chain fatty acids (SCFAs) (acetate and butyrate). In addition, AST significantly decreased the expression of TLR4, MyD88, and p-p65, while increasing the expression of p65. Meanwhile, the expression of inflammatory factors including TNF-*α* and INF-*γ* decreased, while the expression of IL-10 increased. In conclusion, AST reduced OTA-induced cecum injury by regulating the cecum barrier function and TLR4/MyD88/NF-*κ*B signaling pathway.

## 1. Introduction

Mycotoxins are a natural pollutant with a long half-life, which is a global concern [1]. OTA is a common and highly concerned small molecule mycotoxin and organic toxin, which is mainly produced by *Aspergillus* and *Penicillium* [2, 3]. After OTA enters into the body, it can cause serious damage to organs and tissues such as severe hepatotoxicity, nephrotoxicity, neurotoxicity, and immune-toxicity [4–6]. It can also inhibit protein synthesis, leading to damage to the barrier function of the intestine and an increase in membrane permeability [7].

Some previous studies have shown that OTA targets the kidney [8, 9]. However, the intestine is an important barrier for the entry of toxic and harmful substances into the body. Therefore, the intestinal tissues have been used as new targets for the study of mycotoxins [10]. The gut is not only the main organ for the digestion and absorption of nutrients but also the first line of defense against toxic and harmful substances entering into the body [11]. The intestinal barrier function mainly includes the physical barrier, chemical barrier, and microbial barrier.

The epithelial cells and TJ proteins form a physical barrier in the gut. The TJ proteins are mainly composed of claudin, occludin, and ZO-1 families. The normal expression and distribution of TJ proteins play an important role in maintaining the integrity of the intestinal mucosal barrier [12]. The normal expression of connexin has been used as an important marker for intestinal injury [13]. The mucosal surface of the intestine is covered with a thick mucus layer, which is known as a chemical barrier and mainly includes mucinous proteins, antimicrobial peptides, digestive enzymes, and immunoglobulins. These are secreted by cells in the intestinal wall and diluted in order to hydrolyze toxic and harmful substances to prevent damage to the body [14–16]. The intestinal tract has a large number of microorganisms, especially in the cecum and colon, and is known as a microbial barrier. The symbiotic bacteria compete to inhibit pathogens from contacting the surface of epithelial cells of the intestine and maintain the integrity of the barrier [17]. At the same time, some special symbiotic bacteria can produce SCFAs to maintain intestinal health [18]. The mycotoxins can destroy the barrier function of intestinal epithelial cells in both animals and humans and can cause intestinal lesions [19]. The mycotoxins can damage the immune barrier of intestinal mucosa [20]. The mycotoxins also affect the composition and proportion of intestinal flora, thereby affecting the contents of SCFAs and damaging the intestinal microbial barrier [1, 21] .

AST is a natural carotenoid, which is found in various marine organisms [22]. Studies have shown that AST has multiple biological activities such as anti-inflammatory, antiapoptotic, immune regulatory, antitumor, antidiabetic, and liver protection [23, 24]. Some previous studies have shown that AST reduces the serum levels of inflammatory mediators and cytokines and inhibits the activation and reactive oxygen species accumulation of lipopolysaccharide-stimulated RAW264.7 cells [25, 26]. The main purpose of this study was to establish a mouse model in order to study the mitigation effects and possible mechanism of AST on OTA-induced cecum injury.

## 2. Materials and Methods

### 2.1. Animals and Treatments

A total of eighty C57 mice (6 weeks old, 20 ± 2 g) were purchased from Shandong Peng Yue Experimental Animal Breeding Co. Ltd. (Shandong, China). These mice were kept in cages in a specific pathogen-free environment, where the indoor temperature and relative humidity were maintained within the range of 21-23°C and 40-60%, respectively, with light/dark alternation for 12 hours a day. Drinking water was provided throughout the day. After three weeks of acclimation, all the mice were randomly divided into four groups: control group (without OTA), OTA group (5 mg/kg body weight), AST group (100 mg/kg body weight), and AST intervention group (100 mg/kg body weight AST+5 mg/kg body weight OTA). All the treatments were given using oral gavage. AST was purchased from Beijing Solarbio Science & Technology Co. Ltd. (Beijing, China) and dissolved in olive oil [27]. OTA was purchased from LKT Labs, Inc. (St. Paul, MN, USA) and dissolved in 0.1 mol/l NaHCO_3_ [28]. The control group received 0.1 ml of olive oil and then gavage with 0.1 ml NaHCO_3_ after 2 h. The OTA group received 0.1 ml OTA and then 0.1 ml olive oil after 2 h. The OTA group received 0.1 ml AST and then 0.1 ml NaHCO_3_ after 2 h. The AST intervention group received 0.1 ml AST and then 0.1 ml OTA after 2 h. The treatments were administered for seven days with a two-day rest for a cycle, repeated three cycles. The mental state and diet of mice were observed continuously during the experiment. After the end of the tests, 5 mice were randomly selected and the cecum was removed and fixed in 4% paraformaldehyde for histomorphological observation and goblet cell count. Serum was collected for endotoxin detection. Cecal feces was collected for detection of flora, SCFAs, and pH value. Cecum tissues were collected for the detection of inflammatory factors and inflammation-related proteins. This study was approved by the Ethics Committee for Laboratory Animal Care (Animal Ethics Procedures and Guidelines of China) for use by Shenyang Agricultural University, China (Permit No. 264 SYXK2011-0001, 20th October, 2011).

### 2.2. Detection of Serum Endotoxin in Mice

The blood samples collected from mouse eyeballs were placed at room temperature for 2 hours. Then, they were centrifuged at 4°C (2500 rpm/min) for 20 minutes. The supernatant was collected, and the serum endotoxin was detected using an ELISA kit (Jiangsu Baolai Biotechnology Co., Ltd., Jiangsu, China) according to the manufacturer's instructions.

### 2.3. Histopathological Observation of Cecum in Mice

The cecum tissues of mice were stained with hematoxylin and eosin (H&E) (Servicebio, Wuhan, China) and then observed using a Leica DM750 software (Leica DM750, Leica, Beijing, China) to evaluate the morphological changes in the cecum.

### 2.4. Goblet Cell Counts in the Cecum of Mice

Five villi were selected from each section, and PAS (Periodic Acid-Schiff) (Servicebio, Wuhan, China) staining was used to make the goblet cells of mouse cecum to appear purplish red. The number of goblet cells was counted, and their average values were calculated.

### 2.5. Detection of Inflammatory Factors and Endotoxin

The cecum was homogenized using a tissue homogenizer, the cecum suspension was centrifuged at 2500 rpm for 20 minutes, and the supernatants were collected. The IL-10, INF-*γ*, TNF-*α*, and endotoxin were tested according to the manufacturer's instructions of the kit (Jiangsu Baolai Biotechnology Co., Ltd., Jiangsu, China).

### 2.6. Determination of the Chemicals Secreted by the Cecum

The cecum was homogenized using a tissue homogenizer, the cecum suspension was centrifuged at 2500 rpm for 20 minutes, and the supernatants were collected. The MUC1, MUC2, Defa5, and *β*-pD2 were tested according to the manufacturer's instructions of the kit, purchased from Jiangsu Baolai Biotechnology Co., Ltd., (Jiangsu, China).

### 2.7. Determination of the pH Value of Cecum Content [29]

The content of cecum was accurately weighed and then mixed thoroughly according to the ratio of content and distilled water (1 : 9). First, a small amount of liquid from the upper layer of the mixture was dropped on a pH test paper using a glass rod and then compared with the color card immediately in order to estimate the pH range of acid and base. Subsequently, the pH value of cecum content was determined using a two-point calibration method in accordance with the specification of the Shanghai mine PHS-25 digital pH-display meter, purchased from Shanghai Yidian Scientific Instrument Co., Ltd. (Shanghai, China).

### 2.8. Determination of the SCFA Contents in Cecum Tissues

The weight of cecum content was accurately weighed, to which PBS solution was added at a ratio of 1 : 4, and mixed with a vortex mixer for 1 minute. Then, 20 *μ*l of orthophosphate was added and mixed again using oscillation. The mixture was then centrifuged at 4000 rpm/min for 10 minutes. For tests, 1 ml of supernatant was absorbed and passed through a 0.22 m filter membrane. The contents of acetate, propionate, and butyrate were detected using gas chromatography (Agilent GC 6890, USA).

### 2.9. Detection of the Intestinal Flora in Cecum

The DNA was extracted from the cecum content sample using an Omega E.Z.N.A.® stool DNA kit according to the manufacturer's instructions. The V4 variable region of bacteria was amplified by PCR using primer 338f-ACTCCTACGGGAGGCAGCA and 806r-GGACTACHVGGGTWTCTAAT and Thermal cycler (ABI GeneAmp® PCR System 9700, US). The PCR product was then sequenced using Illumina's TruSeq Nano DNA LT Library Prep Kit. After high-throughput sequencing, the data was analyzed.

### 2.10. Western Blotting Analysis

The expressions of TLR4, MyD88, p65 (NF-*κ*B), p-p65 (p-NF-*κ*B), and TJ proteins (occludin, claudin-1, and ZO-1) were detected using western blot. About 0.1 g of the cecum was taken, to which 900 *μ*l of lysate (Solarbio, Beijing, China) was added in order to extract cecum proteins. The extracted protein concentration was quantified using a BCA kit (Solarbio, Beijing, China). The proteins were boiled at 100°C for 10 minutes to denature. The proteins (15 *μ*g) were subjected to 12.5% SDS-PAGE and then transferred to a PVDF (Solarbio, Beijing, China) membrane. The primary antibodies (CST, Boston, MA, USA) were incubated with 5% skim milk powder (Solarbio, Beijing, China), anti-TLR4 (1 : 1000), anti-MyD88 (1 : 1000), anti-p65 (1 : 1000), anti-p-p65 (1 : 1000), anti-claudin-1 (1 : 1000), anti-occludin (1 : 1000), anti-ZO-1 (1 : 1000), and beta-actin (1 : 1000). The secondary antibody (CST, USA) (1 : 10,000) was then incubated at room temperature for 2 hours. ECL (enhanced chemiluminescence) solution (Solarbio, Beijing, China) exposed proteins to the PVDF membrane. GelQuant software was used to detect the gray value of the strip.

### 2.11. Statistical Analysis of Data

All the data in this study was statistically analyzed using SPSS v13.0 and expressed as the mean ± standard deviation (*X* ± SD). The differences were analyzed using one-way ANOVA, and the histogram was drawn using GraphPad Prism v5.0 software. The value below or equal to 0.05 was considered to be statistically significant.

## 3. Results

### 3.1. Effects of OTA and AST on the Daily Feed Intake in Mice

**Figure 1:**
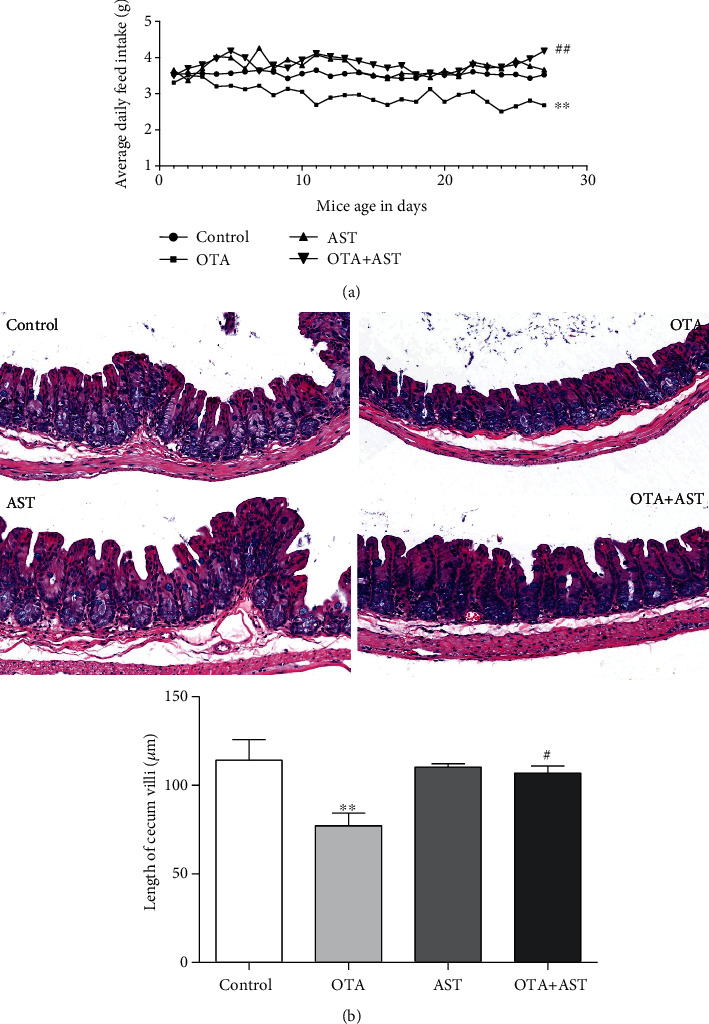
Effects of OTA and AST on the daily feed intake in mice and length of mouse cecum villi. (a) The daily feed intake in mice *n* = 20 mice/group; (b) the length of mouse cecum villi *n* = 5 mice/group (200x), *n* = 5 mice/group. The control, OTA, AST, and OTA+AST represent the control group, OTA group, AST group, and AST intervention group, respectively. In comparison with the control group, ^∗∗^*P* < 0.01 and OTA group; ^#^*P* < 0.05, ^##^*P* < 0.01 were considered to be statistically significant.

Compared with the control group, the average daily feed intake was significantly lower in the OTA group (*P* < 0.01). When the mice were treated with AST, a significant increase in the average daily feed intake was observed (*P* < 0.01) (Figure 1(a)).

### 3.2. Effects of OTA and AST on the Length of Mouse Cecum Villi

The histopathological changes in mouse cecum were observed using H&E staining. The shape of the mouse villi in the control group was normal. As compared to the control group, the mice in the OTA group had significantly shorter cecum villi (*P* < 0.01) and thinner intestinal walls (Figure 1(b)). In comparison with the OTA group, the mice in the AST intervention group had a significant increase in cecum villi (*P* < 0.05) and thick intestinal walls (Figure 1(b)).

### 3.3. Effects of OTA and AST on the Content of Endotoxins in Mouse Serum and Tissue

**Figure 2:**
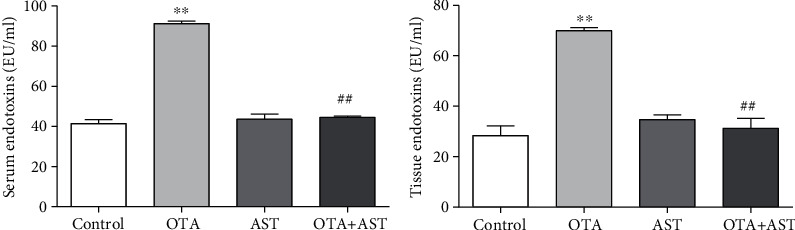
Effects of OTA and AST on the changes in serum endotoxin content in mice, *n* = 5 mice/group. The control, OTA, AST, and OTA+AST represent the control group, OTA group, AST group, and AST intervention group, respectively. In comparison with the control group, ^∗∗^*P* < 0.01 and OTA group; ^##^*P* < 0.01 were considered to be statistically significant.

In comparison with the control group, the endotoxin contents in serum and tissue in the OTA group were significantly higher than that in the control group (*P* < 0.01) (Figure 2), while in the AST intervention group, it was significantly lower than that in the OTA group (*P* < 0.01) (Figure 2).

### 3.4. Effects of OTA and AST on the Chemical Immune Barrier of Cecum

**Figure 3:**
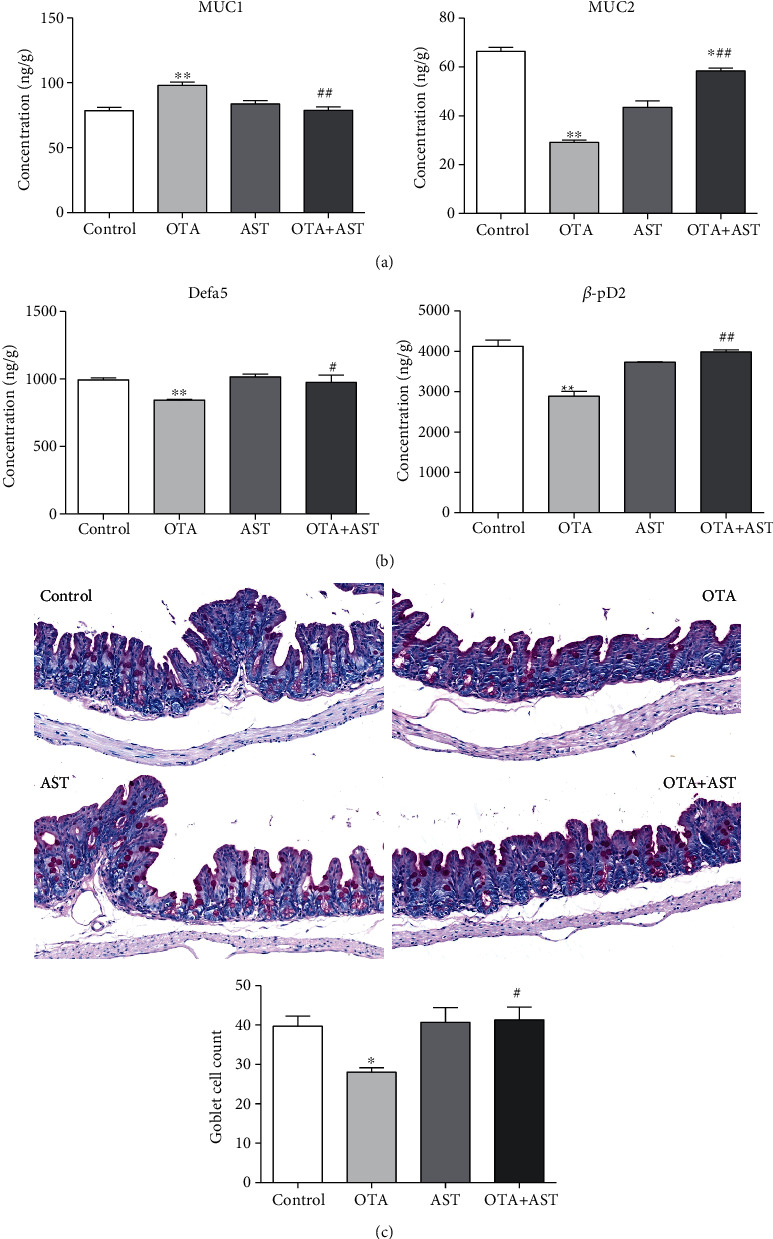
Effects of OTA and AST on the cecum histochemical immune barrier. (a–c) The effects of OTA and AST on the concentrations of MUC1 and MUC2, Defa5, and *β*-pD2 and the number of goblet cells, respectively, in the mouse cecum (200x). The control, OTA, AST, and OTA+AST represent the control group, OTA group, AST group, and AST intervention group, respectively. In comparison with the control group, ^∗^*P* < 0.05 and ^∗∗^*P* < 0.01 and OTA group; ^#^*P* < 0.05, ^##^*P* < 0.01 were considered to be statistically significant.

As compared with the control group, the content of MUC1 in the OTA group increased significantly (*P* < 0.01), while in comparison with the OTA group, the content of MUC1 in the AST intervention group significantly reduced (*P* < 0.01). As compared to the control group, the content of the MUC2 in the OTA group significantly reduced (*P* < 0.01). In comparison with the OTA group, the content of MUC2 significantly increased in the AST intervention group (*P* < 0.01) (Figure 3(a)).

In comparison with the control group, the contents of Defa5 and *β*-pD2 in the OTA group significantly decreased (*P* < 0.01), while in comparison with the OTA group, the content of Defa5 in the AST intervention group increased (*P* < 0.05), and the content of *β*-pD2 in the AST intervention group increased significantly (*P* < 0.01) (Figure 3(b)).

As compared to the control group, the number of goblet cells in the OTA group decreased significantly (*P* < 0.05), while in comparison with the OTA group, it significantly increased in the AST intervention group (*P* < 0.05) (Figure 3(c)).

### 3.5. Effects of OTA and AST on the Protein Content and Concentration of Inflammatory Factors of Cecum Inflammatory Pathway in Mice

**Figure 4:**
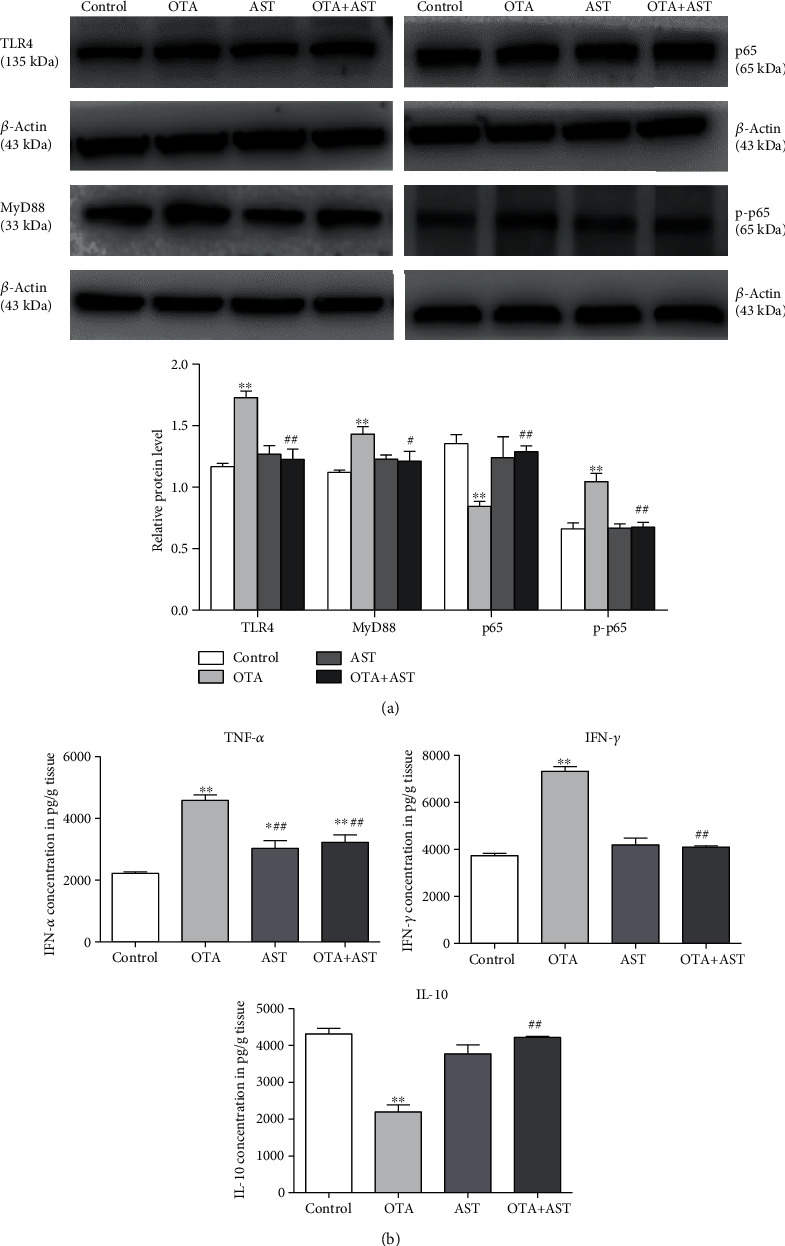
Effects of OTA and AST on the protein content and concentration of inflammatory factors of cecum inflammatory pathway in mice. (a) The effects of OTA and AST on changes in the relative contents of TLR4, MyD88, p65, and p-p65 in mouse cecum. (b) The effects of OTA and AST on changes in the contents of various inflammatory markers (IL-10, IFN-*γ*, and TNF-*α*) in mouse cecum. *n* = 5 mice/group. The control, OTA, AST, and OTA+AST represent the control group, OTA group, AST group, and AST intervention group, respectively. In comparison with the control group, ^∗∗^*P* < 0.01 and OTA group; ^#^*P* < 0.05, ^##^*P* < 0.01 were considered to be statistically significant.

As compared to the control group, the relative contents of TLR4, MyD88, and p-p65 in the OTA group significantly increased (*P* < 0.01), while the relative content of p65 significantly reduced (*P* < 0.01). In comparison with the OTA group, the relative contents of TLR4, p-p65, and MyD88 in the AST intervention group significantly reduced (*P* < 0.01 for TLR4 and p-p65 and *P* < 0.05 for MyD88), while that of p65 significantly increased (*P* < 0.01) (Figure 4(a)).

In comparison with the control group, the contents of IFN-*γ* and TNF-*α* in the OTA group increased significantly (*P* < 0.01) (Figure 4(b)), while that in the AST intervention group decreased significantly (*P* < 0.01) (Figure 4(b)). As compared to the control group, the IL-10 content in the OTA group significantly reduced (*P* < 0.01), while in the AST intervention group, that significantly increased (*P* < 0.01) (Figure 4(b)).

### 3.6. Changes in the Relative Contents of Occludin, Claudin-1, and ZO-1 in Mouse Cecum Induced by OTA and AST

**Figure 5:**
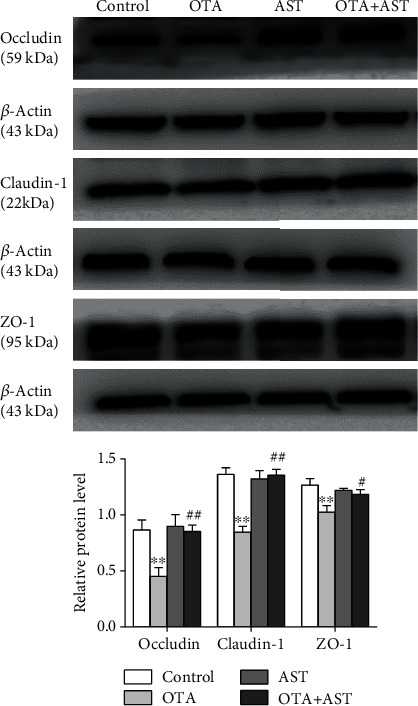
Effect of OTA and AST on the relative contents of occludin, claudin-1, and ZO-1 proteins in TJ proteins in mouse cecum. *n* = 5 mice/group. The control, OTA, AST, and OTA+AST represent the control group, OTA group, AST group, and AST intervention group, respectively. In comparison with the control group, ^∗∗^*P* < 0.01 and OTA group; ^#^*P* < 0.05, ^##^*P* < 0.01 were considered to be statistically significant.

In comparison with the control group, the relative contents of occludin, claudin-1, and ZO-1 in TJ proteins in the mice of the OTA group were significantly reduced (*P* < 0.01). In comparison with the OTA group, the relative contents of occludin, claudin-1, and ZO-1 in the AST intervention group were significantly increased (*P* < 0.01 for occludin and claudin-1 and *P* < 0.05 for ZO-1) (Figure 5).

### 3.7. Effects of OTA and AST on the Content of SCFAs in Mouse Cecum

**Figure 6:**
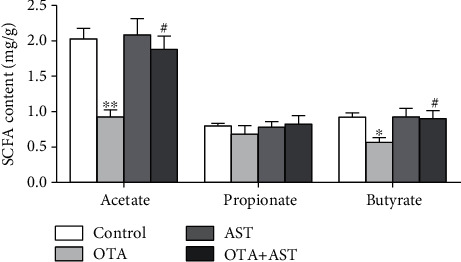
Effects of OTA and AST on the concentrations of SCFAs in mouse cecum. *n* = 5 mice/group. The control, OTA, AST, and OTA+AST represent the control group, OTA group, AST group, and AST intervention group, respectively. In comparison with the control group, ^∗^*P* < 0.05 and ^∗∗^*P* < 0.01 and OTA group; ^#^*P* < 0.05 were considered to be statistically significant.

As compared to the control group, the content of acetate in the OTA group decreased significantly (*P* < 0.01); the content of butyrate decreased significantly (*P* < 0.05). As compared to the OTA group, the contents of acetate and butyrate in the AST intervention group were significantly increased (*P* < 0.05) (Figure 6).

### 3.8. Effects of OTA and AST on Changes of Intestinal Flora in Cecum

#### 3.8.1. Effects of OTA and AST on the *α*-Diversity and pH Value of Cecum Flora in Mice

Alpha diversity reflects the species richness and diversity of samples, including multiple indicators, Chao1, Ace, Shannon, and Simpson. In general, the larger Chao1 or ACE index is, the higher the species abundance will be. The higher the Shannon or Simpson index is, the higher the species diversity is. According to Table 1, as compared to the control group, ACE and Chao1 indices of the OTA group were significantly reduced (*P* < 0.05); in comparison with the OTA group, the ACE index of the AST intervention group significantly increased (*P* < 0.05) and the Chao1 index of the AST intervention group had a trend to increase. In comparison with the control group, the Shannon and Simpson indices of the OTA group had a trend to decrease; in comparison with the OTA group, that of the AST intervention group had a trend to increase. As compared to the control group, the pH value of cecum content in the OTA group was significantly reduced (*P* < 0.01), while in comparison with the OTA group, the pH value of the AST intervention group significantly increased (*P* < 0.01), which are listed in Table 1.

#### 3.8.2. Effects of OTA and AST on the *β*-Diversity of Cecum Flora in Mice

**Figure 7:**
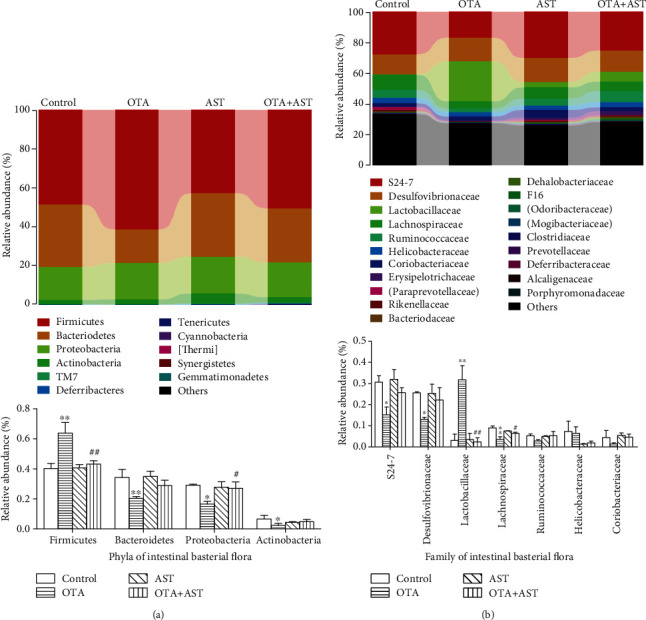
Effects of OTA and AST on the diversity of cecum flora in mice. (a, b) The effects of compositions at the gate and section level, respectively. The control, OTA, AST, and OTA+AST represent the control group, OTA group, AST group, and AST intervention group, respectively. *n* = 5 mice/group. In comparison with the control group, ^∗^*P* < 0.05 and ^∗∗^*P* < 0.01 and OTA group; ^#^*P* < 0.05, ^##^*P* < 0.01 were considered to be statistically significant.

As can be seen from Figure 7(a), the Firmicutes, Bacteroidetes, Proteobacteria, and Actinobacteria were the main phyla of mouse cecum flora. As compared to the control group, the relative abundance of Firmicutes in the OTA group increased significantly (*P* < 0.01), while the relative abundance of Bacteroidetes, Proteobacteria, and Actinobacteria decreased significantly (*P* < 0.01 for Bacteroidetes and *P* < 0.05 for Proteobacteria and Actinobacteria). In comparison with the OTA group, the relative abundance of Firmicutes in the AST intervention group decreased significantly (*P* < 0.01), while that of Proteobacteria increased significantly (*P* < 0.05). The relative abundance of Bacteroidetes and Actinobacteria in AST intervention had a trend to increase (*P* > 0.05).

It can be seen from Figure 7(b) that the effects of OTA and AST on change in the composition of cecum content at the family level mainly included significant changes in the relative abundance of S24-7, Desulfovibrionaceae, Lactobacillaceae, and Lachnospiraceae. As compared to the control group, the relative abundance of Lactobacillaceae in the OTA group increased by about ten times (*P* < 0.01), and the relative abundance of Desulfovibrionaceae, S24-7, and Lachnospiraceae decreased significantly (*P* < 0.05 for Desulfovibrionaceae and S24-7 and *P* < 0.01 for Lachnospiraceae). In comparison with the OTA group, the relative abundance of Lactobacillaceae in the AST intervention group significantly decreased (*P* < 0.01); the relative abundance of Lachnospiraceae increased significantly (*P* < 0.05).

## 4. Discussion

The intestinal tract is not only exposed to a variety of microorganisms but also exposed to many toxic and harmful substances. Therefore, the maintenance of the integrity of the upper cortex is critical in order to inhibit pathogens from entering the intestinal tract and maintain the balance of nutrients' exchange [30, 31]. The test results showed that after OTA administration, the length of villi shortened and the thinning of the intestinal wall occurred. Villi length and mucosal thickness in the cecum of mice were observed to had a significant recovery compared with the control group after the administration of AST, which was in agreement with a previous study [19].

It has been hypothesized that most serum endotoxin is derived from the gut, since breakdown of the intestinal barrier would possibly contribute to the translocation of products of bacteria [32]. And endotoxin can induce intestinal inflammation and increase permeability in each intestinal section [33]. The experimental results indicated that after the addition of AST, the endotoxin contents in serum and tissue were significantly reduced. A study in colonic epithelial cells has shown that endotoxin can decrease the expression of TJ proteins (ZO-1 and claudin-1) and increase paracellular permeability [34, 35]. As an endotoxin sensor, once activated, TLR4 can generate inflammatory response and cause cecum damage [36], which shows that endotoxin is closely related to TLR4 pathways and inflammation.

The TLRs are one of the most important pattern recognition receptors and play a key role in the induction of inflammatory responses and production of inflammatory mediators [37]. The TLR4 is a key proinflammatory response regulator, which causes the phosphorylation of mitogen-activated protein kinases (MAPKs) through a chain of MAPKs and activates the NF-*κ*B signaling pathway. Stimulation of the TLR4/Myd88 signaling pathway induces NF-*κ*B p65 phosphorylation and then translocates from the cytoplasm into the nucleus, leading to inflammation and generation of inflammatory cytokines (TNF-*α* and INF-*γ*) [38–40]. Studies have suggested that macrophages in the lamina propria of the intestine may produce large amounts of TNF-*α*. Overproduction of TNF-*α* has been reported to cause direct damage to the integrity of intestinal mucosa and epithelial function [41]. In the present study, the experimental results suggested that OTA promoted the contents of NF-*κ*B-related proteins and increased the expression levels of proinflammatory cytokines (TNF-*α*, INF-*γ*), which were consistent with the previous study results that the OTA induced the inflammatory response by activating the TLR4/MyD88/NF-*κ*B pathway [39]. However, the above indicators were inhibited after the addition of AST, and AST could increase the expression of IL-10. IL-10 is an anti-inflammatory cytokine, which can reduce inflammatory response and enhance local immunity in intestinal mucosa [42].

It is widely known that TJ proteins are vital components of the intestine structure to maintain the permeability and homeostasis of intestinal epithelial cells [43]. Occludins and claudins are core TJ proteins, which control the epithelial structure and permeability of the intestine, while ZO-s (ZO-1, ZO-2, ZO-3) are frame-forming proteins, which connect actin cytoskeleton to two or more transmembrane proteins [44]. Research has shown that OTA can aggravate deoxynivalenol-induced intestinal barrier dysfunction by disrupting TJ proteins and activating the NF-*κ*B signaling pathway, indicating that TJ proteins are closely linked to the NF-*κ*B signaling pathway [45]. It has already been demonstrated that cytokines, including IFN-*γ* and TNF-*α*, are associated with the downregulation of TJ proteins [46]. The western blot analysis showed that the relative contents of occludin, claudin-1, and ZO-1 in the AST intervention group significantly increased as compared to the OTA group. These results suggest that AST could protect the structural integrity of cecum by increasing contents of TJ proteins and decreasing expressions of inflammatory factors to suppress the activation of the NF-*κ*B inflammatory pathway.

The gastrointestinal tract is regarded as the largest immune organ in the body, which plays a core role in the regulation of immune homeostasis [47]. The mycotoxins can harm the function of the immune system in animals and may increase their susceptibility to infectious diseases and morbidity [48]. The surface of the intestine is covered with a layer of mucus, containing mucin, defensins, slgA, etc., which are important components of the innate immune system [46]. The mucin provides protection against invasive pathogens by preventing the direct contact of pathogenic bacteria with the surface of epithelial cells [49], and it can supplement the nutrition of bacteria in the gut as an energy source [50]. Loss of mucin produced in goblet cells and TJ proteins can contribute to the leakage of endotoxin. Reduction of MUC2 can compromise the integrity of the intestine [51]. Our results showed that the decrease of MUC2 was related to the decrease of goblet cells. The increase in the MUC1 level could prevent pathogens from entering into the body. However, AST suppressed these changes by improving intestinal barrier function. Moreover, defensins are natural peptides implicated in resisting intestinal pathogen infection [52]. *β*-pD2 can competitively inhibit the activation of the NF-*κ*B signaling pathway by binding to TLR4, thereby repressing the release of downstream inflammatory cytokines [16]. Defa5 was shown to alleviate mouse colitis via downregulating the expression of proinflammatory cytokines and inhibiting the NF-*κ*B pathway [53]. Our data showed that the protective effect of AST on intestinal barrier function occurs through inhibiting the OTA-induced reduction of Defa5 and *β*-pD2 expression. This finding was consistent with the above results.

The microbiota of the hindgut is the basis for the maintenance of barrier function and fermentation capacity [54]. The microbiota plays an important role in defense against pathogens and in the maintenance of intestinal health [55]. The diversity of the gut microbiota reflects the complication of the species within the gut microbiota. In our study, the OTA reduced the *α*-diversity of intestinal flora and increased the proportion of Firmicutes and Lactobacillaceae. A study has shown that the abundance of Firmicutes has an inverse association with the number of intestinal pathogens [56]. According to the results of our test, the changes of the proportion of Firmicutes were mainly caused by the changes in the proportion of lactobacillus, which indicated that the lactobacillus was a key detoxifying bacteria in the OTA group [57]. Our data was consistent with a previous study [58]. Moreover, OTA reduced the abundance of Lachnospiraceae, which was subsequently recuperated after supplementation with AST. Lachnospiraceae families are capable of fermenting various substrates to butyrate [59]. Administration of AST increased the diversity of gut microbiota and ameliorated main gut flora (especially Lactobacillaceae and Lachnospiraceae), which was in agreement with a previous study [60].

In addition, most studies have confirmed that SCFAs have positive physiological functions in the body, and they are produced mainly by gut microorganisms through anaerobic fermentation. In a study, mice were orally treated with acetate and propionate, and their findings showed that acetate can inhibit ethanol-induced gastric injury via suppressing gastric oxidation, inflammation, and apoptosis and promoting mucin expression [61]. Butyrate can provide energy for the growth of intestinal epithelial cells, enhance intestine mucosal integrity, and inhibit inflammation [62]. Microbial butyrate may contribute to the restoration of the TJ barrier via upregulating the protein levels of ZO-1 [63], claudin-3, and claudin-4 [64]. And it can also improve the intestinal permeability to reduce the entry of endotoxin into the blood, significantly decreased TLR4 and its downstream protein Myd88, and further inactivates the translocation of NF-*κ*B into the nucleus and the induction of proinflammatory gene expression such as TNF-*α* and INF-*γ* [65]. Lactobacillus with numerous advantageous effects produces lactate, which can increase butyrate production in feces [66]. The intestinal pH is primarily affected by the number of fermentable carbohydrates entering the large intestine and is used as an indicator for intestinal health [67]. The results of this study showed that the contents of acetate and butyrate were significantly reduced in the AST intervention group. Our results were consistent with the protective effects of AST on intestinal mucosal immune disorders caused by cyclophosphamide [60].

Overall, our results suggested that the cecum barrier under OTA-induced injury could be ameliorated by AST.

## 5. Conclusions

This study showed that the AST had a protective effect on OTA-induced cecum injury in mice. The protective mechanism of the AST lies in the barrier function of the cecum and TLR4/MyD88/NF-*κ*B inflammatory signaling pathway. This study provides a new theoretical basis and research direction for the clinical application of AST in the prevention and treatment of intestine injuries.

## Figures and Tables

**Table 1:** Effects of OTA and AST on the *α*-diversity of cecum flora and pH value of cecum content. *n* = 5 mice/group.

Item	Control	OTA	AST	OTA+AST
Chao1	2368.01 ± 225.01	1831.76 ± 421.86^∗^	2211.56 ± 179.78	2204.39 ± 29.76
ACE	2219.26 ± 96.27	1710.98 ± 392.79^∗^	2280.93 ± 220.07	2278.3 ± 76.61^#^
Shannon	7.81 ± 0.16	6.45 ± 1.12	7.78 ± 0.39	7.51 ± 0.09
pH value	7.37 ± 0.12	6.56 ± 0.28^∗∗^	7.41 ± 0.06	7.42 ± 0.17^##^

Note: data has been presented as the mean ± S.D. In comparison with the control group, ^∗^*P* < 0.05 and ^∗∗^*P* < 0.01 and OTA group; ^#^*P* < 0.05, ^##^*P* < 0.01 were considered to be statistically significant.

## Data Availability

The data used to support the findings of this study are included within the article.
